# Chronic Oral Administration of Mineral Oil Compared With Corn Oil: Effects on Gut Permeability and Plasma Inflammatory and Lipid Biomarkers

**DOI:** 10.3389/fphar.2021.681455

**Published:** 2021-08-16

**Authors:** Elsbet J. Pieterman, Hans M. G. Princen, Annica Jarke, Ralf Nilsson, Anders Cavallin, Linnéa Bergenholm, Marcus Henricsson, V. Sashi Gopaul, Rahul Agrawal, Steven E. Nissen, Eva Hurt-Camejo

**Affiliations:** ^1^ The Netherlands Organisation for Applied Scientific Research (TNO), Metabolic Health Research, Leiden, Netherlands; ^2^ Advanced Drug Delivery, Pharmaceutical Sciences, BioPharmaceuticals R&D, AstraZeneca, Gothenburg, Sweden; ^3^ Early Cardiovascular Renal and Metabolism, BioPharmaceuticals R&D, AstraZeneca, Gothenburg, Sweden; ^4^ Global Cardiovascular Renal and Metabolism, BioPharmaceuticals R&D, AstraZeneca, Gothenburg, Sweden; ^5^ Department of Cardiovascular Medicine and Cleveland Clinic Coordinating Center for Clinical Research, Cleveland Clinic, Cleveland, OH, United States

**Keywords:** APOE*3-Leiden.CETP mice, cholesterol, fatty acids, intestinal permeability, triglycerides

## Abstract

We investigated the effects of chronic oral administration of mineral oil, versus corn oil as control, on intestinal permeability, inflammatory markers, and plasma lipids in APOE*3-Leiden.CETP mice. Mice received mineral oil or corn oil 15 or 30 μL/mouse/day for 16 weeks (15 mice/group). Intestinal permeability was increased with mineral versus corn oil 30 µL/day, shown by increased mean plasma FITC-dextran concentrations 2 h post-administration (11 weeks: 1.5 versus 1.1 μg/ml, *p* = 0.02; 15 weeks: 1.7 versus 1.3 μg/ml, *p* = 0.08). Mean plasma lipopolysaccharide-binding protein levels were raised with mineral versus corn oil 30 µL/day (12 weeks: 5.8 versus 4.4 μg/ml, *p* = 0.03; 16 weeks: 5.8 versus 4.5 μg/ml, *p* = 0.09), indicating increased intestinal bacterial endotoxin absorption and potential pro-inflammatory effects. Plasma cholesterol and triglyceride concentrations were decreased with mineral oil, without affecting liver lipids among treated groups. Fecal neutral sterol measurements indicated increased fecal cholesterol excretion with mineral oil 30 µL/day (+16%; *p* = 0.04). Chronic oral administration of mineral oil in APOE*3-Leiden.CETP mice increased intestinal permeability, with potential pro-inflammatory effects, and decreased plasma cholesterol and triglyceride levels. Our findings may raise concerns about the use of mineral oil as a placebo in clinical studies.

## Introduction

Results from recent clinical trials assessing the effects of omega-3 fatty acids on triglyceride levels and cardiovascular risk show that the placebo mineral oil arms had increased levels of non-high-density lipoprotein cholesterol, C-reactive protein, and apolipoprotein (Apo)B (Bays et al., 2011; Ballantyne et al., 2012; Bhatt et al., 2019), which are markers for inflammation and coronary artery disease risk (Ridker et al., 1997; Ridker et al., 2000; Ference et al., 2017), raising concerns about the use of mineral oil as a placebo (Haslam and Prasad, 2019; Kastelein and Stroes, 2019). Little information is available on the biological effects of orally administered mineral oil (European Food Safety Authority (EFSA) Panel on Contaminants in the Food Chain (CONTAM), 2012). As a short-term laxative, mineral oil is given for a few days to alleviate constipation by lubricating the feces and intestinal wall (Candy et al., 2011; Gordon et al., 2016). Mineral oil can affect the absorption of some compounds, such as fat-soluble vitamins, in the gastrointestinal tract (Steigmann et al., 1952; Prescriber’s Digital Reference, 2020). Our recent work in wild-type mice has indicated that short-term chronic oral administration of mineral oil as part of a Western-type diet does not affect the absorption of standard-of-care statins (Gopaul et al., 2021). However, chronic administration of mineral oil tended to cause direct, low-grade inflammation, shown by raised levels of both serum amyloid A (SAA), a marker for acute-phase inflammation, and circulating L-selectin-expressing (CD62L^high^) B cells (Gopaul et al., 2021).

As a follow-up, the current study aimed to investigate further the potential low-grade inflammation brought about by chronic administration of mineral oil compared with corn oil, given as part of a Western-type diet. The starting point for the design of our study was the use of two different placebo treatments (mineral oil and corn oil) in clinical studies with omega-3 fatty acids, which may result in different clinical outcomes. Therefore, we compared the effects of these two placebo treatments in our study in mice. We hypothesized that chronic oral administration of mineral oil would increase gut permeability, causing leakage of lipopolysaccharide (LPS; also known as endotoxin) from the gut into the circulation, leading to increased levels of inflammation markers. An additional aim was to measure the effect of mineral oil on plasma lipids in view of the increased levels of non-high-density lipoprotein cholesterol observed in clinical studies (Bays et al., 2011; Ballantyne et al., 2012; Bhatt et al., 2019) and the reported effect of mineral oil on the absorption of fat-soluble compounds (Steigmann et al., 1952; Prescriber’s Digital Reference, 2020). We used ApoE*3-Leiden cholesteryl ester transfer protein (APOE*3-Leiden.CETP) mice, which are a well-established translational animal model with a human-like lipoprotein metabolism (Westerterp et al., 2006; Kühnast et al., 2015a). APOE*3-Leiden.CETP mice are suitable for studying the effects of different treatments on plasma cholesterol and triglyceride levels, atherosclerosis, metabolic syndrome, and non-alcoholic steatohepatitis (Ason et al., 2014; Kühnast et al., 2015a; Kühnast et al., 2015b; Pouwer et al., 2020; van den Hoek et al., 2020; van den Hoek et al., 2014; Westerterp et al., 2006).

## Materials and Methods

### Formulation

The composition of mineral oil used in this study was 99.7% (w/w) mineral oil light + 0.3% (w/w) alpha-tocopherol (antioxidant). The composition of corn oil used was 99.7% (w/w) corn oil + 0.3% (w/w) alpha-tocopherol, consistent with the corn oil placebo used in the Outcomes Study to Assess Statin Residual Risk Reduction With Epanova in High CV Risk Patients With Hypertriglyceridemia (STRENGTH) (Nicholls et al., 2018; Nicholls et al., 2020). Mineral oil light was purchased from Sigma-Aldrich (United States) (CAS number: 8042–47–5). It is a highly refined petroleum mineral oil consisting of a complex combination of saturated hydrocarbons (aliphatic hydrocarbons, viscosity 15.3 centistokes at 40°C), having carbon numbers predominantly in the range C15 through C50 (European Chemicals Agency (ECHA), 2021b). Alpha-tocopherol was purchased from Sigma-Aldrich (Germany). The corn oil + alpha-tocopherol mixture was extracted from clinical sample placebo corn oil capsules used in STRENGTH (Nicholls et al., 2018; Nicholls et al., 2020), supplied by Catalent (Germany; corn oil supplied by Henry Lamotte Oils GMbH [CAS number: 8001–30–7]). The corn oil fatty acid composition is comprised of primarily linoleic acid and oleic acid (European Chemicals Agency (ECHA), 2021a).

### Mice

Female APOE*3-Leiden.CETP mice were obtained from the Netherlands Organisation (TNO), Metabolic Health Research, Leiden, Netherlands. Female APOE*3-Leiden.CETP mice were used because they are more susceptible to cholesterol-containing diets by having higher plasma cholesterol and triglyceride levels than male APOE*3-Leiden.CETP mice and thus develop more pronounced atherosclerotic lesions (van Vlijmen et al., 1996). Mice were housed in Makrolon cages (maximum five animals per cage) in conventional animal rooms at approximately 21°C with a 12 h light/12 h dark cycle and provided access to water *ad libitum*. The welfare of the mice was maintained in accordance with the general principles governing the use of animals in experiments of the European Communities (2010/63/EU) and Dutch legislation (The Experiments on Animals Act, 2014).

### Experimental Design

Mice (8–12 weeks of age) were fed a semi-synthetic, Western-type diet containing 0.15% cholesterol, 15% cacao butter, 1% corn oil, 40.5% sucrose, 10% starch, 20% casein, 5.95% cellulose, 2% choline, 0.2% methionine, and 5.35% vitamin and mineral mix (all w/w) (Ssniff, Soest, Germany). After a 3-weeks run-in period, the mice were matched by age, body weight, and plasma cholesterol and triglycerides levels, and were divided into four groups of 15 animals each. Groups were assigned to one of four regimens for 16 weeks, given orally as admixture to the Western-type diet: mineral oil 15 µL/mouse/day; mineral oil 30 µL/mouse/day; corn oil 15 µL/mouse/day; or corn oil 30 µL/mouse/day.

Body weight was assessed at 0, 4, 8, 11, 12, 15, and 16 weeks. Feces were collected at 15 weeks over two consecutive 3-day periods. Blood samples were collected via tail vein bleedings after 4 h of fasting at 0, 4, 8, 12, and 16 weeks. To determine intestinal permeability at 11 and 15 weeks, mice received 900 mg/kg body weight of FITC-dextran 4 kDa (average molecular weight 3–5 kDa), followed by collection of blood samples 2 h later. All animals were euthanized by CO_2_ inhalation at 16 weeks. Blood for fluorescence-activated cell sorting analysis was collected by heart puncture. Cecal contents were collected for assessment of endotoxin levels and liver tissue was collected for lipid analysis.

### Dosing Rationale

The mineral oil dosing rationale was guided by simplified body surface area and intestinal surface area calculations to translate the human dosing of 4 ml mineral oil used in the clinical trials (Bays et al., 2011; Ballantyne et al., 2012; Bhatt et al., 2019) to mice. The following body surface area calculation, as accepted by the US Food and Drug Administration (FDA) (Nair and Jacob, 2016), was used: human dose × mouse body weight divided by human body weight × 12.3; thus, 4 ml/day in an 80 kg human equates to 15 μL/day for a 24 g mouse. The intestinal surface area calculation was based on the difference in intestinal surface area between mice and humans, as follows: human dose × intestinal surface area in mouse divided by intestinal surface area in human (Casteleyn et al., 2010); thus, 4 ml/day in a human equates to 30 μL/day for a mouse. We used both approaches in the present study by administering 15 and 30 μL per mouse per day, consistent with our previous study in which we investigated the effect of mineral oil on the absorption of statins.

### Sample Analysis

#### Intestinal Permeability

Intestinal permeability was determined at 11 and 15 weeks. Plasma samples taken 2 h after oral administration of FITC-dextran were analyzed for amount of fluorescence and plotted on a FITC-dextran standard curve to calculate FITC-dextran concentrations in the plasma. Endotoxin levels in cecum were assessed at 16 weeks using the *Limulus* Amoebocyte Lysate assay (Lonza) according to the manufacturer’s instructions.

#### Plasma Inflammatory Markers

Plasma levels of endotoxin, LPS-binding protein (LBP), and SAA were determined at 0, 4, 8, 12, and 16 weeks. Endotoxin levels were determined using the *Limulus* Amoebocyte Lysate assay (Lonza, Basel, Switzerland), LBP was measured using the mouse LBP enzyme-linked immunosorbent assay kit (Hycult Biotech, Uden, Netherlands), and SAA levels were measured using the mouse SAA enzyme-linked immunosorbent assay kit (Tridelta Development Ltd., Maynooth, Ireland), according to the manufacturers’ instructions.

Plasma levels of alanine aminotransferase and aspartate aminotransferase were determined at 0, 4, 8, 12, and 16 weeks in samples pooled per group using the Reflotron system with appropriate strips (Boehringer Mannheim, Mannheim, Germany). Fluorescence-activated cell sorting analysis of peripheral blood mononuclear cells at 16 weeks was performed using the following antibodies: CD11b-FITC, CD62L-PerCP-Cy5.5, CD19-APC-eFluor780, CD44-PE, Ly6C-APC, CD3-eFluor450, and CD11c-PE-Cy7 (all from Affymetrix eBioscience, San Diego, CA, United States). Plasma levels of cytokines were determined for selected samples at 0, 8, 12, and 16 weeks using the mouse V-PLEX pro-inflammatory panel 1 mouse kit (Meso Scale Discovery, Rockville, MD, United States), which consists of the cytokines interferon-gamma, interleukin (IL)1-beta, IL-2, IL-4, IL-5, IL-6, KC/GRO, IL-10, IL-12p70, and tumor necrosis factor-alpha.

#### Plasma and Liver Lipids

Plasma total cholesterol levels, triglyceride levels and pooled lipoprotein profiles were determined at 0, 4, 8, 12, and 16 weeks. Total cholesterol and triglyceride concentrations were measured using the CHOD-PAP and TG GPO-PAP analyzer kits, respectively (Roche/Hitachi, Roche Diagnostics, Rotkreuz, Switzerland). Pooled lipoprotein profiles were assessed by fast protein liquid chromatography (ÄKTA). Cholesterol and phospholipid profiles in the pooled samples were measured using the CHOD-PAP kit (Roche/Hitachi, Roche Diagnostics, Rotkreuz, Switzerland) and the Phospholipids Assay Kit (INstruchemie, Delfzijl, Netherlands), respectively. Liver free cholesterol, cholesterol esters, and triglycerides levels were determined in eight mice per group from the 30 µL/mouse/day groups as described previously (Havekes et al., 1987).

#### Fecal Neutral Sterols

Neutral sterols in feces were analyzed as described previously (Post et al., 2003). Feces were collected per cage at 15 weeks, over two consecutive time periods of 3 days, in the 30 µL/mouse/day groups (*n* = 8 samples per group).

#### Plasma and Liver Fatty Acids

Plasma total fatty acids were analyzed for controls (baseline [0 weeks], 10 samples) and in mice fed with corn oil 30 µL/mouse/day for 12–16 weeks (22 samples) or mineral oil 30 µL/mouse/day for 12–16 weeks (26 samples). The plasma samples were spiked with tricosanoic acid (C23:0) and extracted using the BUME method (Löfgren et al., 2012). Fatty acid methyl esters were produced by acid-catalyzed transesterification and were analyzed using an Agilent 7890A gas chromatograph coupled to an Agilent 5975C mass spectrometer. For separation, an Agilent DB-23 column was used. In total, 17 fatty acids were analyzed and quantified against the C23:0 fatty acid internal standard.

Liver fatty acids were extracted at 16 weeks from homogenized liver samples (500 µg protein per sample) by a Bligh and Dyer protocol (Bligh and Dyer, 1959). Approximately 10–20 mg samples of the different diets were used for dietary fatty acid composition analyses. After derivatization to fatty acid methyl esters, both liver and dietary fatty acids were analyzed by gas chromatography using a CP-Sil 88 column (Agilent Technologies Inc., Santa Clara, CA, United States) as described previously (Princen et al., 1995). In total, 17 fatty acids were analyzed.

### Statistical Analyses

Statistical analyses were performed using SPSS Statistics Version 25.0 (IBM Corporation). Values are represented as means ± standard deviations (SDs). Area under the concentration–time curve (AUC) was calculated as the sum of the average levels during *t* = 0–4, 4–8, 8–12, and 12–16 weeks, multiplied by the number of weeks for each time frame (i.e., 4 weeks). A Shapiro–Wilk test was used to check whether data were normally distributed. Depending on normal distribution, significance of differences between the 15 µL/mouse/day dosing groups and the 30 µL/mouse/day dosing groups was measured either parametrically using a Student’s *t*-test for independent samples or non-parametrically using a Mann Whitney *U*-test for independent samples. *p-*values < 0.05 were considered statistically significant and values between 0.05 and 0.10 were considered to be trending towards statistical significance.

## Results

### Animals

The mice appeared normal, behaved normally, and showed no signs of discomfort during the study. Food intake remained similar between groups. At 0 weeks, mean body weights for the four groups ranged from 21.2 g (SD: 1.4) to 21.4 g (SD: 1.4), and at 16 weeks they ranged from 24.3 g (SD: 2.0) to 25.6 g (SD: 2.3). Body weights remained similar between all four groups during the 16-weeks study period with the exception of a lower mean body weight at 8 weeks in the 15 µL mineral oil group than in the 15 µL corn oil group (22.6 g [SD: 1.5] versus 24.2 g [2.2]; *p* = 0.021). Liver weight, heart and spleen weight, intestinal weight, and intestinal length were similar between the four groups at the end of the 16-weeks study period, with no statistically significant differences between the mineral oil and the corn oil groups.

### Intestinal Permeability

**FIGURE 1:**
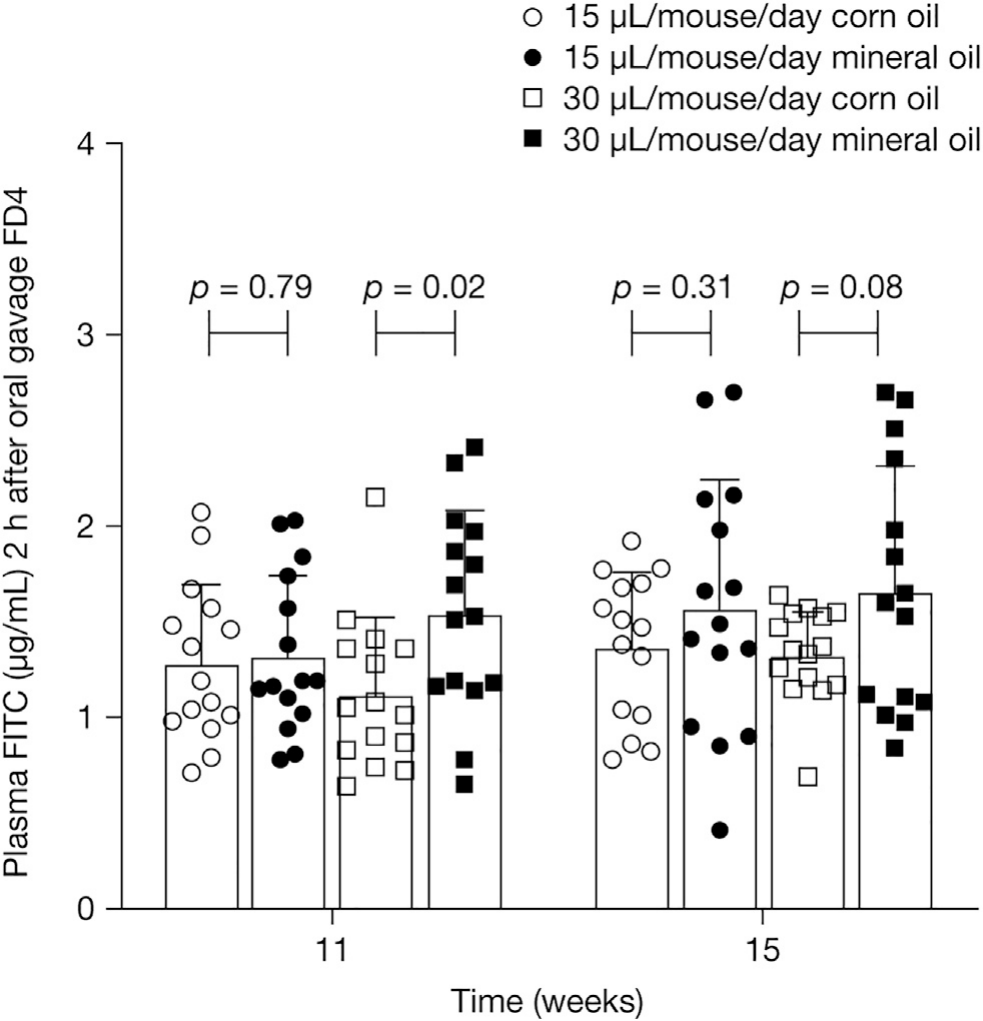
Mean (SD) plasma concentrations of FITC-dextran 4 kDa 2 h after oral administration of FITC-dextran at 11 and 15 weeks. *p*-values were calculated using independent samples *t*-test. FD4, FITC-dextran 4 kDa; SD, standard deviation.

Plasma FITC-dextran concentrations 2 h after oral administration of FITC-dextran were statistically significantly higher in the 30 µL mineral oil group than in the 30 µL corn oil group at 11 weeks (mean [SD]: 1.5 [0.5] versus 1.1 [0.4] µg/ml; *p* = 0.02) and trended towards statistical significance at 15 weeks (mean [SD]: 1.7 [0.7] versus 1.3 [0.2] µg/ml; *p* = 0.08) (Figure 1). Endotoxin levels in cecum fluid at 16 weeks were not significantly affected by either dose of mineral oil, compared with corn oil (mean [SD] endotoxin values [*10^3^ EU/mL] for mineral oil versus corn oil, 15 µL/mouse/day: 6.7 [2.1] versus 6.3 [1.7], *p* = 0.62; 30 µL/mouse/day: 6.5 [2.6] versus 6.8 [2.3], *p* = 0.77).

### Plasma Inflammatory Markers

**FIGURE 2:**
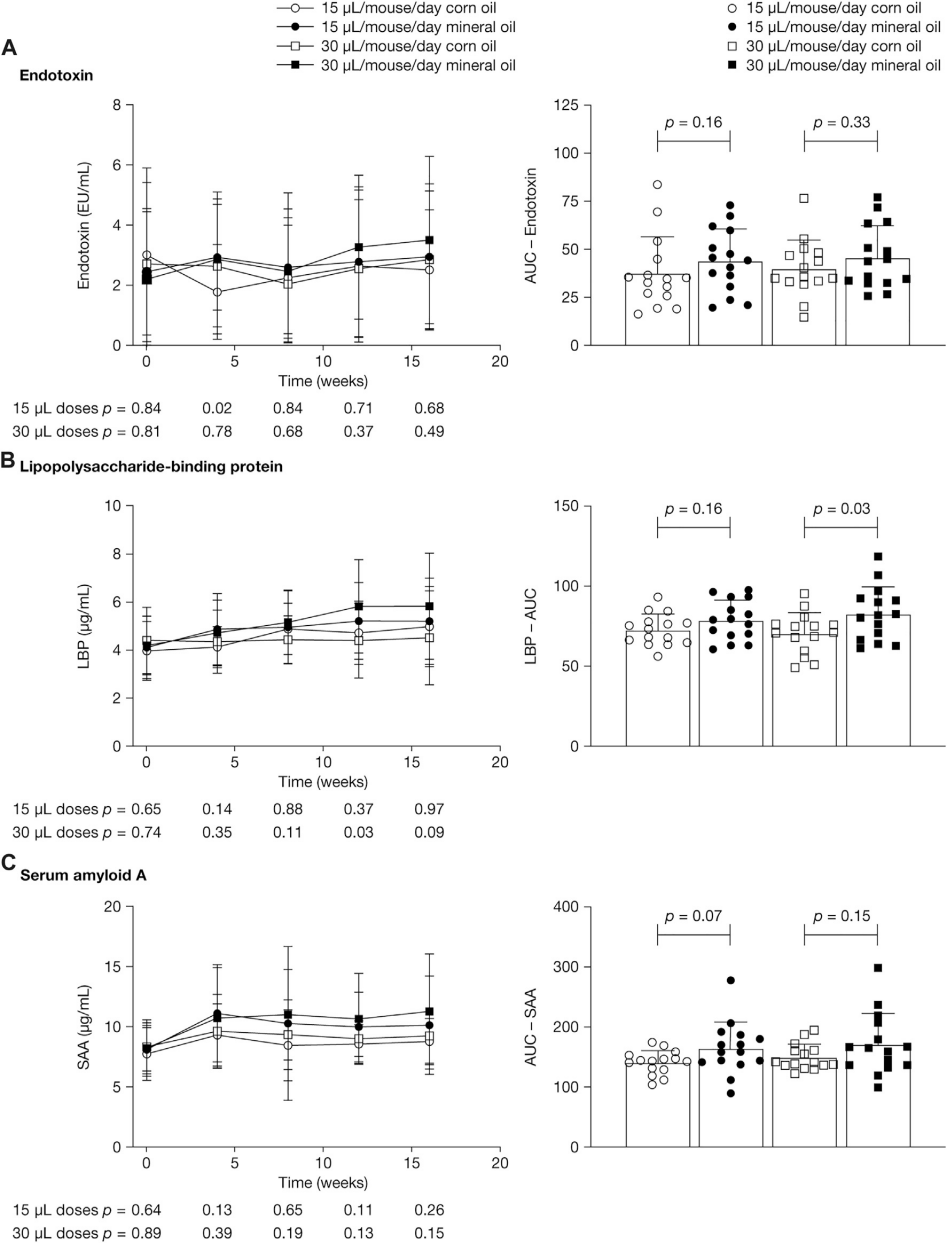
Mean (SD) plasma concentrations at 0, 4, 8, 12, and 16 weeks (left panels) and area under the concentration–time curve (right panels) for **(A)** endotoxin, **(B)** lipopolysaccharide-binding protein, and **(C)** serum amyloid A. *p*-values were calculated using independent samples *t*-test. AUC, area under the concentration–time curve; LBP, lipopolysaccharide-binding protein; SAA, serum amyloid A; SD, standard deviation.

Figure 2 shows mean plasma concentrations at 0, 4, 8, 12, and 16 weeks and AUC values for endotoxin, LBP, and SAA. LBP is an acute-phase protein that binds to bacterial LPS and is necessary for the rapid acute-phase response to LPS; SAA is an acute-phase protein mainly produced by the liver. Endotoxin levels varied widely, with large within-group SDs; mean values ranged between 2.0 EU/mL and 3.0 EU/mL and did not differ significantly between groups at the time points assessed (Figure 2A). Levels of endotoxin in plasma were approximately 3,000 times lower than in cecum fluid.

Mean (SD) LBP concentrations were significantly higher in the 30 µL mineral oil group than in the 30 µL corn oil group at 12 weeks (5.8 [1.9] versus 4.4 [1.6] µg/ml; *p* = 0.03) and trended towards statistical significance at 16 weeks (mean [SD]: 5.8 [2.2] versus 4.5 [2.0] µg/ml; *p* = 0.09) (Figure 2B). At 4 weeks and 8 weeks, there were no statistically significant differences in LBP concentrations between the mineral oil and the corn oil groups. The AUC for LBP was significantly higher in the 30 µL mineral oil group than in the 30 µL corn oil group (*p* = 0.03). For SAA concentrations, there was a trend towards statistically significant differences between the 15 µL mineral oil group and the 15 µL corn oil group for AUC (*p* = 0.07) (Figure 2C).

Pooled plasma levels for alanine aminotransferase and aspartate aminotransferase at 0, 4, 8, 12, and 16 weeks were similar between the mineral oil and the corn oil groups (Supplementary Table S1). Mean plasma composition at 16 weeks for peripheral blood mononuclear cells and for T-cell, B-cell, and monocyte subsets were not statistically significantly different between the mineral oil and the corn oil groups (Supplementary Figure S1). The cytokines successfully analyzed using the V-plex panel were IL-2, IL-5, IL-6, KC/GRO, IL-10, and tumor necrosis factor-alpha. No statistically significant differences in plasma levels of these cytokines were observed between the mineral oil and the corn oil groups (Supplementary Table S2). The levels of the remaining cytokines were too low for any conclusions to be drawn.

### Plasma and Liver Lipids

**FIGURE 3:**
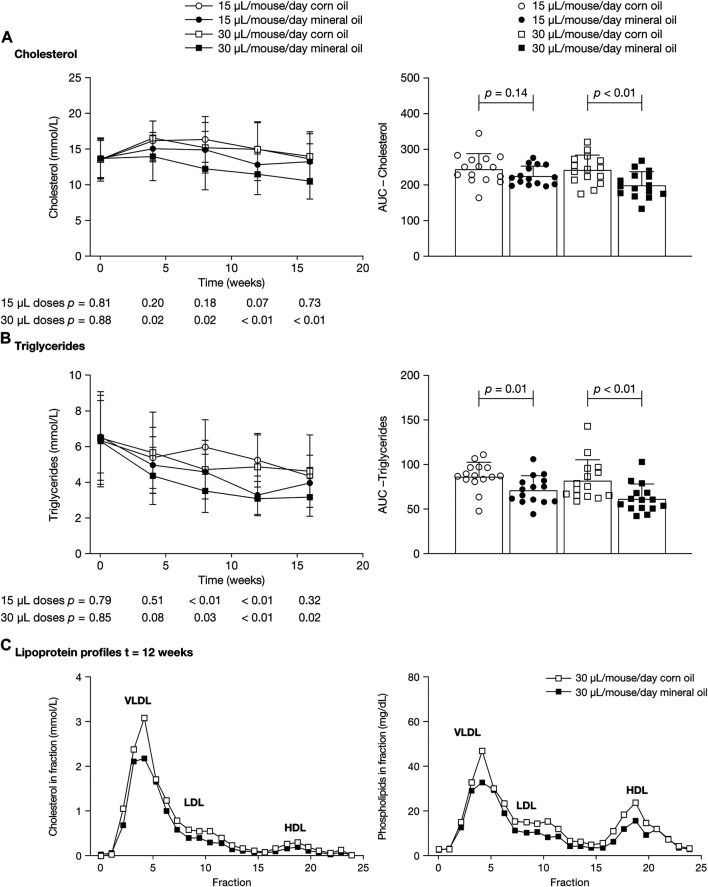
Mean (SD) plasma concentrations at 0, 4, 8, 12, and 16 weeks (left panels) and area under the concentration–time curve (right panels) for **(A)** cholesterol and **(B)** triglycerides. *p*-values were calculated using independent samples *t*-test. **(C)** Lipoproteins from pooled plasma samples were size-fractionated and analyzed by fast protein liquid chromatography. Profiles across the fractions from samples at 12 weeks are shown for cholesterol (left panel) and phospholipids (right panel). AUC, area under the concentration–time curve; HDL, high-density lipoprotein; LDL, low-density lipoprotein; SD, standard deviation; VLDL, very low-density lipoprotein.

Cholesterol concentrations were significantly lower in the 30 µL mineral oil group than in the 30 µL corn oil group at the four post-baseline time points (mean [SD], mmol/L – 4 weeks: 14.0 [3.4] versus 16.6 [2.4], *p* = 0.02; 8 weeks: 12.3 [2.9] versus 15.3 [3.5], *p* = 0.02; 12 weeks: 11.6 [2.8] versus 15.1 [3.7], *p* < 0.01; 16 weeks: 10.6 [2.5] versus 14.1 [3.4], *p* < 0.01) (Figure 3A). The AUC for cholesterol was significantly lower in the 30 µL mineral oil group than in the 30 µL corn oil group (*p* < 0.01). There were no statistically significant differences in plasma cholesterol values between the 15 µL mineral oil and corn oil groups.

Mean triglyceride concentrations were statistically significantly lower in the 30 µL mineral oil group than in the 30 µL corn oil group at 8, 12, and 16 weeks (mean [SD], mmol/L – 8 weeks: 3.5 [1.2] versus 4.7 [1.6], *p* = 0.03; 12 weeks: 3.1 [1.0] versus 4.8 [1.8], *p* < 0.01; 16 weeks: 3.2 [1.1] versus 4.6 [2.0], *p* = 0.02), and were significantly lower in the 15 µL mineral oil group than in the 15 µL corn oil group at 8 and 12 weeks (mean [SD], mmol/L – 8 weeks: 4.6 [1.0] versus 6.1 [1.6], *p* < 0.01; 12 weeks: 3.3 [1.1] versus 5.2 [1.5], *p* < 0.01) (Figure 3B). The AUC for triglycerides was significantly lower in the mineral oil group than in the corn oil group, both for the 30 µL dose (*p* < 0.01) and for the 15 µL dose (*p* = 0.01).

Lipoprotein profiles in pooled plasma showed lower cholesterol content in very low-density lipoprotein and low-density lipoprotein in the mineral oil groups than in the corn oil groups. Profiles from the 30 µL dose groups at 12 weeks are shown in Figure 3C.

**FIGURE 4:**
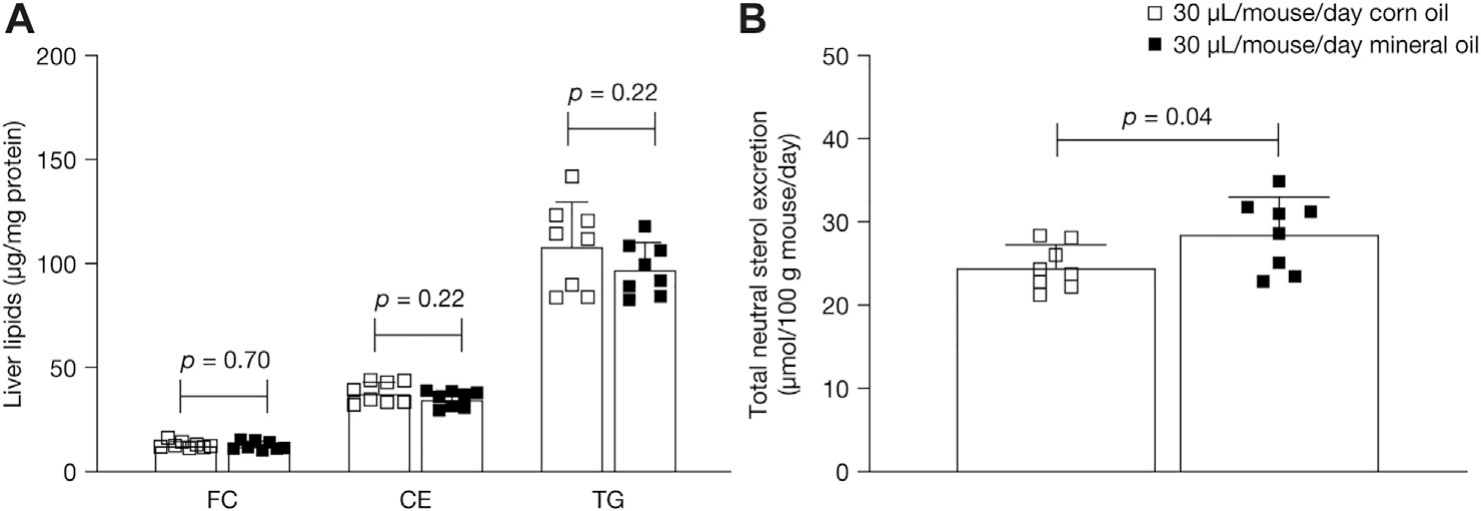
Mean (SD) **(A)** liver free cholesterol, cholesterol esters, and triglycerides at 16 weeks and **(B)** total neutral sterols excretion in feces at 15 weeks. *p*-values were calculated using independent samples *t*-test. CE, cholesterol esters; FC, liver free cholesterol; SD, standard deviation; TG, triglycerides.

Lipid content in the liver in the 30 µL dose groups was measured to assess whether the changes in plasma lipids after administration of mineral oil compared with corn oil resulted in changes in hepatic cholesterol and triglycerides. Mean liver free cholesterol, cholesterol ester, and triglyceride levels at 16 weeks did not differ statistically significantly between the mineral oil group and the corn oil group (Figure 4A).

### Fecal Neutral Sterols

Fecal excretion of neutral sterols was measured in the 30 µL dose groups to examine if mineral oil affected the intestinal uptake of cholesterol (Figure 4B). Mean total neutral sterol concentrations were significantly higher in the 30 µL mineral oil group than in the 30 µL corn oil group (+16%; mean [SD]: 28.5 [4.4] versus 24.5 [2.7] µmol/100 g mouse/day, *p* = 0.04). Composition analysis showed that, as a proportion of total neutral sterols, the mineral oil group had significantly lower levels of cholesterol (mean [SD]: 94.9% [0.3%] versus 95.2% [0.3%], *p* = 0.05) and significantly higher levels of cholestanol (3.0% [0.3%] versus 2.6% [0.2%], *p* < 0.01) than the corn oil group, with no significant difference in lathosterol levels (2.1% [0.2%] versus 2.2% [0.1%], *p* = 0.13).

### Plasma and Liver Fatty Acids

**FIGURE 5:**
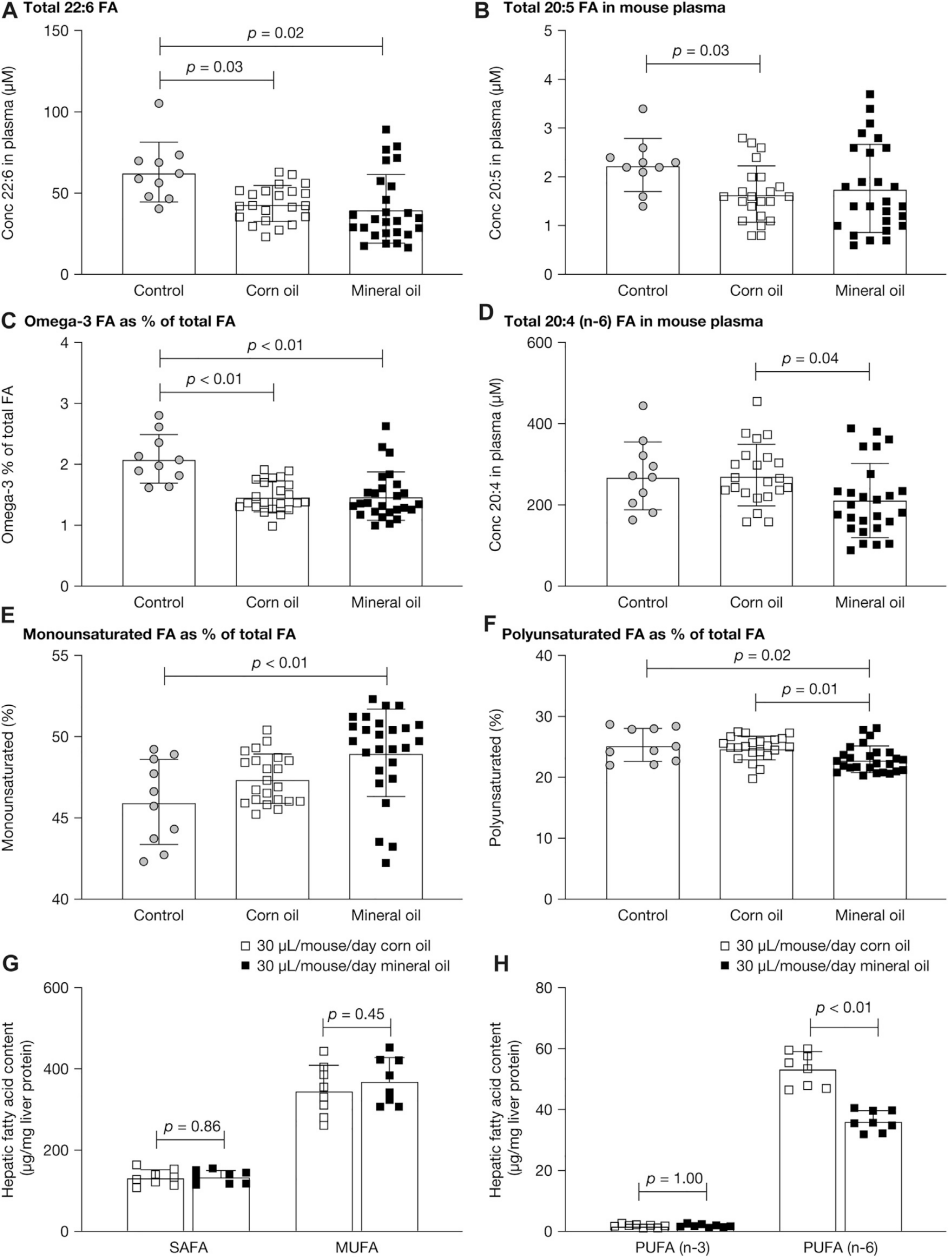
Plasma and liver fatty acids in control mice and mice fed with corn oil 30 µL/mouse/day or mineral oil 30 µL/mouse/day for 12 or 16 weeks: **(A)** total 22:6 (docosahexaenoic acid) fatty acid concentration in plasma, **(B)** total 20:5 (EPA) fatty acid concentration in plasma, **(C)** Omega-3 index in plasma, **(D)** total 20:4n6 (arachidonic acid) fatty acid concentration in plasma, **(E)** percent monounsaturated fatty acids in plasma, **(F)** percent polyunsaturated fatty acids in plasma, **(G)** SAFA and MUFA in liver, and **(H)** PUFA n-3 and n-6 in liver. *p*-values were calculated using independent samples *t*-test. EPA, eicosapentaenoic acid; FA, fatty acid; MUFA, monounsaturated fatty acid; PUFA, polyunsaturated fatty acid; SAFA, saturated fatty acid.

The fatty acid composition of lipids was measured in plasma and liver because mineral oil had reduced the plasma lipid level and had affected intestinal absorption. Figures 5A–F show the plasma fatty acid results for untreated controls and mice given mineral oil 30 µL/mouse/day or corn oil 30 µL/mouse/day for 12–16 weeks for the parameters with statistically significant between-group differences. The mean plasma concentration of 22:6 fatty acids (docosahexaenoic acid) and the proportion of omega-3 fatty acids as a percentage of total fatty acids in plasma (the Omega-3 index) were statistically significantly decreased in both the mineral oil group and the corn oil group, compared with the control group (all *p* < 0.05). The mean plasma concentration of 20:5 fatty acid (eicosapentaenoic acid) was statistically significantly decreased in the corn oil group (*p* = 0.03), but not in the mineral oil group, compared with the control group. The mean plasma concentration of 20:4 fatty acid (n-6, arachidonic acid) was statistically significantly lower in the mineral oil group than in the corn oil group. For the remaining 14 of the 17 plasma fatty acid parameters assessed, there were no statistically significant between-group differences (Supplementary Table S3).

As a proportion of total fatty acids, monounsaturated fatty acids were statistically significantly increased in the mineral oil group compared with the control group (*p* < 0.01) and polyunsaturated fatty acids were statistically significantly decreased in the mineral oil group compared with the corn oil group (*p* = 0.01) and the control group (*p* = 0.02). The proportions of saturated fatty acids were similar between groups.

There were no statistically significant between-group differences for saturated or monounsaturated fatty acids in liver (Figure 5G). The level of polyunsaturated fatty acids was decreased (by −31%) in the mineral oil compared with the corn oil group, due to a significantly lower level of n-6 polyunsaturated fatty acids (*p* < 0.01) (Figure 5H). By contrast, there were no statistically significant between-group differences in levels of n-3 polyunsaturated fatty acids.

Full results for liver fatty acid content are listed in Supplementary Table S4. Compared with the 30 µL corn oil group, the 30 µL mineral oil group had statistically significantly increased levels of palmitoleic acid (C16:1; +41%, *p* < 0.01) and significantly decreased levels of C14:1 (−53%, *p* < 0.01), C18:0 (−10%, *p* = 0.03), C18:2 cis (−37%, *p* < 0.01), C20:0 (−52%, *p* < 0.01), C20:1 (−14%, *p* = 0.04), C20:4n6 (arachidonic acid; −23%, *p* < 0.01), C24:0 (−24%, *p* = 0.04), and C24:1 (−44%, *p* < 0.01). No statistically significant between-group differences were observed for oleic acid (C18:1 cis) or palmitic acid (C16:0), which are the most abundant liver fatty acids, or for C12:0, C14:0, C18:1 trans, C18:2 trans, C20:5n3 (eicosapentaenoic acid), or C22:6n3 (docosahexaenoic acid).

Supplementary Table S5 lists the fatty acid content in the diet of the 15 and 30 µL corn oil and mineral oil groups. Levels of C18:2 cis and n-6 polyunsaturated fatty acids were noticeably higher in the diet of the 30 µL corn oil group than in that of the 30 µL mineral oil group.

## Discussion

This study was conducted to investigate potential effects of mineral oil on intestinal permeability, inflammation, and plasma lipids in APOE*3-Leiden.CETP mice, a mouse model with a human-like lipoprotein metabolism. APOE*3-Leiden.CETP mice are more suitable than wild-type mice as a preclinical model to study the effects of different treatments on plasma lipid levels and inflammatory conditions, such as atherosclerosis and non-alcoholic steatohepatitis (Ason et al., 2014; Kühnast et al., 2015a; Kühnast et al., 2015b; Pouwer et al., 2020; van den Hoek et al., 2020; van den Hoek et al., 2014; Westerterp et al., 2006). We hypothesized that chronic oral administration of mineral oil would increase intestinal permeability, causing leakage of endotoxin from the gut into the circulation and leading to increased inflammation over time. Our results showed increased levels of 4 kDa FITC-dextran in the plasma of mice given it orally with chronic administration of 30 µL mineral oil compared with corn oil, supporting the hypothesis that chronic oral administration of mineral oil increases intestinal permeability.

The observed increase in intestinal permeability appears to have resulted in an increased uptake of endotoxin from the gut microflora in the mice given mineral oil, measured by raised LBP levels in plasma. No statistically significant differences in plasma endotoxin levels were observed between the mineral oil and the corn oil groups, potentially because of inherent assay limitations (Gnauck et al., 2016). Endotoxin levels in cecum fluid were almost 3000-times higher than plasma endotoxin levels and were not significantly affected by either dose of mineral oil, indicating that there was no increased lysis of intestinal flora. In Syrian hamsters, which, compared with wild-type mice, have a more human-like lipoprotein profile, endotoxin has been shown to increase low-density-lipoprotein-cholesterol levels (Feingold et al., 1993). SAA levels in mice given mineral oil in the present study showed a trend towards higher levels than in mice given corn oil, suggesting increased cytokine-mediated inflammation with chronic mineral oil administration, in line with our recent work in wild-type mice (Gopaul et al., 2021). Liver function does not seem to have been affected, as there were similar levels of alanine aminotransferase and aspartate aminotransferase in the mineral oil and the corn oil groups.

The doses of mineral oil used in our study are representative of the 4 ml/day dose used in clinical trials (Bays et al., 2011; Ballantyne et al., 2012; Bhatt et al., 2019). The 4 ml/day mineral oil dose in humans translates to 30 μL/mouse/day, based on the difference in intestinal surface area between humans and mice (human dose × intestinal surface area in mouse divided by intestinal surface area in human) (Casteleyn et al., 2010). Alternatively, based on the simplified body surface area calculation as accepted by the FDA (Nair and Jacob, 2016), a mineral oil dose of 4 ml/day in a human equates to 15 μL/mouse/day for a 24 g mouse. Both doses were assessed in our study.

The potential pro-inflammatory effects seen in our study in the intestine of mice administered mineral oil are of particular interest considering previous data showing that fractions in mineral oil are absorbed by the intestine and may accumulate in tissues (Chuberre et al., 2019). Mineral oil consists of a mix of compounds that are differentially absorbed in the intestine depending on carbon chain length and can accumulate in tissues due to low biotransformation rate (Chuberre et al., 2019; European Food Safety Authority (EFSA), 2012). An association between exposure to mineral oils (e.g., occupational exposure via inhalation and skin exposure) and increased risk of developing autoimmune diseases, such as rheumatoid arthritis, has been reported in epidemiological studies (Chuberre et al., 2019; European Food Safety Authority (EFSA), 2012). In addition, infiltration of mineral oil used subcutaneously for cosmetic purposes has been observed to lead to chronic granulomatous inflammation with increases in pro-inflammatory cytokines and to local and systemic manifestations that resemble autoimmune diseases such as systemic lupus erythematosus, rheumatoid arthritis, and systemic sclerosis (Vera-Lastra et al., 2018). These data indicate that mineral oil is not inert and that compounds in mineral oil have systemic availability.

In the current study, plasma cholesterol and triglyceride levels were decreased in the mineral oil group compared with the corn oil group, whereas there were no differences in the levels of liver lipids. Fecal analysis showed that there was an increased excretion of neutral sterols of +16% in the 30 µL mineral oil group compared with the 30 µL corn oil group. As a proportion of total fecal neutral sterols, cholesterol was lower in the mineral oil group than in the corn oil group, whereas cholestanol was increased with mineral oil. Cholestanol is formed from the biohydrogenation of cholesterol by the intestinal flora and may be indicative of a longer residence time in the gut. Together, these findings indicate that the increased fecal excretion of cholesterol in the mineral oil group leads to lower plasma bioavailability compared with the corn oil group. Increased fecal excretion of cholesterol has also been reported in a study that assessed the effect of mineral oil on intestinal cholesterol absorption in rats (Karvinen et al., 1955). That study also showed increased excretion of saponifiable fat (although without reporting *p-*values) (Karvinen et al., 1955), indicating increased excretion of fatty acids, which may provide an explanation for the decrease in plasma triglyceride levels observed in the current study. The intake of mineral oil in the rat study was about 6% by weight of the daily diet, whereas the dose used in the present study is 6- to 12-fold lower and translates better to the human intake.

The decreases in plasma cholesterol and triglycerides in the absence of accumulation of these lipids in the liver are considered as beneficial from a metabolic perspective and with respect to development of atherosclerosis. We have previously shown that, in APOE*3-Leiden or APOE*3-Leiden.CETP mice, similarly as in humans (Kühnast et al., 2015a; Ference et al., 2017), cholesterol lowering by dietary means (e.g., Volger et al., 2001), or after administration of hypolipidemic drugs (e.g., statins, fibrates, ezetimibe and the novel PCSK9 and ANGPTL3 inhibitors) results in reduced atherogenesis (van De Poll et al., 2001; Kooistra et al., 2006; Ason et al., 2014; Gierman et al., 2014; Kühnast et al., 2015b; Pouwer et al., 2020). For these drugs the response is similar as in patients both with respect to the magnitude and the direction of the changes and the dose, taking into account an about 12-fold higher metabolism (Nair and Jacob, 2016; Pouwer et al., 2019). However, these favorable effects may be counteracted by the consequences of increased intestinal permeability potentially leading to pro-inflammatory effects, which have been reported to pose an increased cardiovascular risk in humans (Ridker et al., 1997; Ridker et al., 2000). It should be noted that decreased plasma cholesterol and triglyceride levels as observed in the present study are not found in clinical studies with mineral oil as placebo treatment, on the contrary increased levels of non-high-density lipoprotein cholesterol and ApoB have been reported in clinical investigations (Bays et al., 2011; Ballantyne et al., 2012; Bhatt et al., 2019).

Differences between the mineral and the corn oil groups in the proportion of polyunsaturated fatty acids in total fatty acid from plasma and liver are likely to have been caused predominantly by the dietary addition of corn oil, which is rich in the n-6 polyunsaturated fatty acid C18:2 (Sacks et al., 2017).

A review article on mineral oil as placebo in REDUCE-IT and other clinical studies concluded that even if mineral oil effects were real, they would be small and would not impact study conclusions (Olshansky et al., 2020). REDUCE-IT showed an increase in the mineral oil placebo groups for levels of non-high-density lipoprotein cholesterol, C-reactive protein, and ApoB (Bhatt et al., 2019). Increases in the levels of these three biomarkers were not found in the corn oil placebo group in the STRENGTH trial (Nicholls et al., 2020). Achieved levels or change in levels of eicosapentaenoic acid and docosahexaenoic acid were associated with neither benefit nor harm in the STRENGTH trial, suggesting that supplementation with these fatty acids in high-risk cardiovascular patients is neutral and that, thus, the choice of placebo may have an important role in determining outcome for trials of omega-3 fatty acids (Nissen et al., 2021). During the initial submission for approval of the icosapent ethyl studied in REDUCE-IT and during review of the REDUCE-IT trial, the FDA had raised concerns about the use of mineral oil as a placebo, but judged in both instances that there was insufficient evidence to conclude that toxicity from the use of mineral oil as a placebo substantially altered outcomes (FDA, 2019). There is FDA agreement with mineral oil as a placebo (FDA, 2019), and studies in which mineral oil was administered, with corresponding biomarker effects, are provided in documentation to the FDA (FDA Briefing Document, 2019). The decision by the FDA was made before completion of the STRENGTH trial.

Future work could involve use of a more advanced disease model, for example mice on a high fat diet, containing a higher amount of saturated fat (45 kcal%) than used in the current study (36 kcal%), to mimic obesity and insulin resistance in the Western world. Such a high fat diet is known to aggravate intestinal permeability in mice (Mueller et al., 2021), which may be further worsened by mineral oil. Future work could also assess where in the body mineral oil accumulates and whether (and how) long-term mineral oil administration affects tissue composition, local cytokine levels in the intestine and liver, and the composition of the gut microbiota.

In conclusion, we show that chronic oral administration of mineral oil in a mouse model with human-like lipoprotein metabolism caused an increase in intestinal permeability, potentially leading to pro-inflammatory effects, and caused a decrease in plasma cholesterol and triglyceride levels. The findings may raise concerns about the use of mineral oil as a placebo in clinical studies.

## Data Availability

The original contributions presented in the study are included in the article/Supplementary Material, further inquiries can be directed to the corresponding author.

## Abbreviations

Apo Apolipoprotein; APOE*3-Leiden.CETP ApoE*3-Leiden cholesteryl ester transfer protein; AUC Area under the concentration–time curve; CD62L Circulating L-selectin-expressing; FDA US Food and Drug Administration; IL Interleukin; LBP Lipopolysaccharide-binding protein; LPS Lipopolysaccharide; SAA Serum amyloid A; SD Standard deviation; STRENGTH Outcomes Study to Assess Statin Residual Risk Reduction With Epanova in High CV Risk Patients With Hypertriglyceridemia; TNO The Netherlands Organisation
