# Supplementary figures and images for: Chronic Oral Administration of Mineral Oil Compared With Corn Oil: Effects on Gut Permeability and Plasma Inflammatory and Lipid Biomarkers
Source: Front Pharmacol. 2021 Aug 16;12:681455. doi: 10.3389/fphar.2021.681455 (PMC8415260; doi:10.3389/fphar.2021.681455)

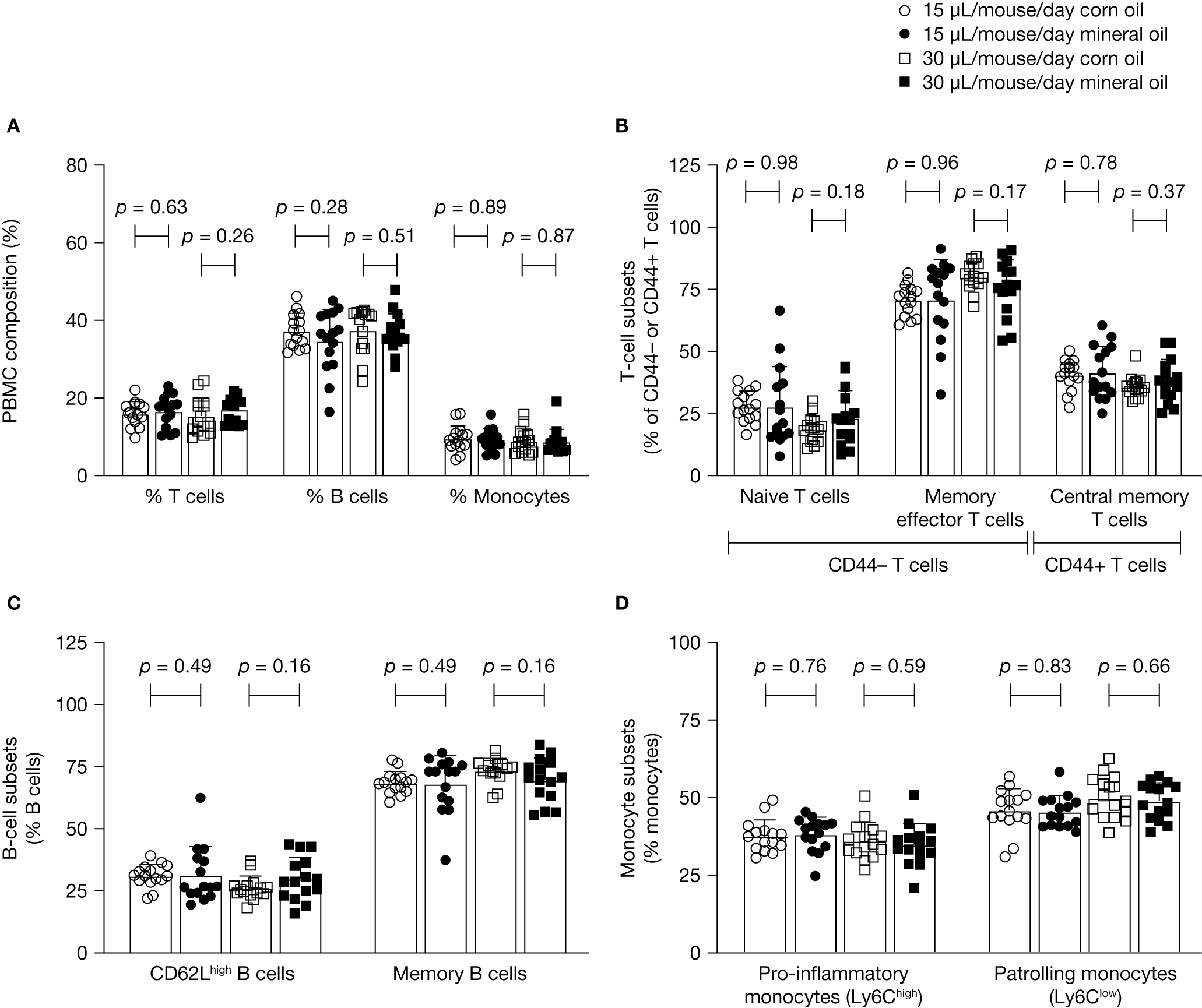

Supplement: Supplementary file 1 [file Image1.TIF]
