# Supplementary material for: Chronic Oral Administration of Mineral Oil Compared With Corn Oil: Effects on Gut Permeability and Plasma Inflammatory and Lipid Biomarkers
Source: Front Pharmacol. 2021 Aug 16;12:681455. doi: 10.3389/fphar.2021.681455 (PMC8415260; doi:10.3389/fphar.2021.681455)
Supplement: Supplementary file 2 [file DataSheet1.docx]

**Supplementary Table S1.** Pooled plasma ALT and AST levels in mice fed with mineral oil 30 µg/mouse/day or corn oil 30 µg/mouse/day for 16 weeks.

|  | **Time, weeks** | | | | |
| --- | --- | --- | --- | --- | --- |
|  | **0** | **4** | **8** | **12** | **16** |
| **ALT, U/L** |  |  |  |  |  |
| Corn oil 15 µL/mouse/day | 48 | 53 | 41 | 31 | 58 |
| Corn oil 30 µL/mouse/day | 46 | 41 | 39 | 32 | 54 |
| Mineral oil 15 µL/mouse/day | 50 | 55 | 29 | 36 | 51 |
| Mineral oil 30 µL/mouse/day | 62 | 46 | 35 | 43 | 53 |
| **AST, U/L** |  |  |  |  |  |
| Corn oil 15 µL/mouse/day | 128 | 139 | 146 | 102 | 163 |
| Corn oil 30 µL/mouse/day | 133 | 114 | 131 | 121 | 159 |
| Mineral oil 15 µL/mouse/day | 140 | 125 | 111 | 113 | 130 |
| Mineral oil 30 µL/mouse/day | 112 | 117 | 116 | 122 | 136 |

Data are absolute values (U/L, 37°C) from plasma samples pooled per group.

ALT, alanine aminotransferase; AST, aspartate aminotransferase; U, units.

**Supplementary Table S2.** Mean ± SD plasma cytokine levels of mice fed with mineral oil 15 or 30 µg/mouse/day or fed with corn oil 15 or 30 µg/mouse/day for 16 weeks.

| [**Analyte**](https://azcollaboration-my.sharepoint.com/personal/kvzs408_astrazeneca_net/Documents/MigratedMyDocs/CVRM/Projects/NNMT/Biomarkers%202020/Biobank%20docs/AZDoc0028375%20checkout.docx?web=1)**, pg/mL** | **Weeks** | **Corn oil 15 µL/mouse/day group** | | **Corn oil 30 µL/mouse/day group** | | **Mineral oil 15 µL/mouse/day group** | | **Mineral oil 30 µL/mouse/day group** | |
| --- | --- | --- | --- | --- | --- | --- | --- | --- | --- |
|  |  | **Mean** | **SD** | **Mean** | **SD** | **Mean** | **SD** | **Mean** | **SD** |
| **IFN-gamma** | 0 | 0.40 | 0.53 | 0.10 | 0.10 | 0.20 | 0.21 | 0.08 | 0.04 |
|  | 8 | 0.08 | 0.12 | 0.13 | 0.08 | 0.17 | 0.26 | 0.64 | 1.17 |
|  | 12 | 0.09 | N/A^a^ | –^b^ |  | 0.07 | 0.04 | –^b^ |  |
|  | 16 | 0.10 | 0.10 | 0.20 | 0.21 | 0.08 | 0.04 | 0.47 | 0.89 |
| **IL-1-beta** | 0 | 0.05 | 0.06 | 0.23 | 0.31 | 0.00 | N/A^a^ | 0.05 | 0.04 |
|  | 8 | 0.12 | 0.16 | 0.08 | 0.10 | 0.05 | 0.05 | 0.03 | 0.04 |
|  | 12 | 0.01 | N/A^a^ | –^b^ |  | 0.01 | 0.00 | –^b^ |  |
|  | 16 | 0.23 | 0.31 | 0.00 | N/A^a^ | 0.05 | 0.04 | 0.19 | 0.31 |
| **IL-2** | 0 | 0.91 | 0.34 | 0.40 | 0.24 | 0.24 | 0.08 | 0.43 | 0.20 |
|  | 8 | 0.24 | 0.12 | 0.24 | 0.11 | 0.23 | 0.13 | 0.26 | 0.14 |
|  | 12 | 0.36 | N/A^a^ | –^b^ |  | 0.30 | 0.09 | –^b^ |  |
|  | 16 | 0.40 | 0.24 | 0.24 | 0.08 | 0.43 | 0.20 | 0.59 | 0.54 |
| **IL-4** | 0 | 0.06 | 0.03 | < 0.42 |  | 0.13 | 0.10 | 0.02 | N/A^a^ |
|  | 8 | < 0.42 |  | < 0.42 |  | 0.23 | N/A^a^ | < 0.42 |  |
|  | 12 | < 0.42 |  | –^b^ |  | <0,42 |  | –^b^ |  |
|  | 16 | < 0.42 |  | 0.13 | 0.10 | 0.02 | N/A^a^ | 0.39 | N/A^a^ |
| **IL-5** | 0 | 3.40 | 1.54 | 4.36 | 2.41 | 3.89 | 1.07 | 6.93 | 7.22 |
|  | 8 | 2.08 | 0.77 | 1.99 | 0.47 | 4.20 | 3.51 | 2.12 | 1.06 |
|  | 12 | 5.42 | N/A^a^ | –^b^ |  | 4.69 | 1.41 | –^b^ |  |
|  | 16 | 4.36 | 2.41 | 3.89 | 1.07 | 6.93 | 7.22 | 3.15 | 0.80 |
| **IL-6** | 0 | 3.96 | 1.64 | 6.43 | 4.34 | 3.61 | 0.90 | 5.63 | 2.28 |
|  | 8 | 3.98 | 1.69 | 7.35 | 4.81 | 4.47 | 0.73 | 5.00 | 2.27 |
|  | 12 | 5.11 | N/A^a^ | –^b^ |  | 7.25 | 8.07 | –^b^ |  |
|  | 16 | 6.43 | 4.34 | 3.61 | 0.90 | 5.63 | 2.28 | 6.97 | 3.43 |
| **KC/GRO** | 0 | 46.47 | 24.30 | 88.23 | 61.28 | 42.82 | 13.74 | 65.71 | 18.91 |
|  | 8 | 85.63 | 34.94 | 77.48 | 28.05 | 64.73 | 29.29 | 48.23 | 16.86 |
|  | 12 | 67.75 | N/A^a^ | –^b^ |  | 66.14 | 32.45 | –^b^ |  |
|  | 16 | 88.23 | 61.28 | 42.82 | 13.74 | 65.71 | 18.91 | 77.37 | 16.23 |
| **IL-10** | 0 | 15.19 | 5.42 | 21.58 | 5.72 | 17.60 | 5.88 | 23.33 | 5.93 |
|  | 8 | 16.29 | 3.83 | 18.67 | 4.21 | 20.11 | 8.14 | 11.81 | 2.56 |
|  | 12 | 25.73 | N/A^a^ | –^b^ |  | 26.29 | 4.33 | –^b^ |  |
|  | 16 | 21.58 | 5.72 | 17.60 | 5.88 | 23.33 | 5.93 | 15.99 | 2.08 |
| **IL-12p70** | 0 | < 7.63 |  | < 7.63 |  | 8.04 | N/A^a^ | < 7.63 |  |
|  | 8 | < 7.63 |  | < 7.63 |  | < 7.63 |  | < 7.63 |  |
|  | 12 | 3.96 | N/A^a^ | –^b^ |  | 4.37 | N/A^a^ | –^b^ |  |
|  | 16 | < 7.63 |  | 8.04 | N/A^a^ | < 7.63 |  | 14.73 | 13.02 |
| **TNF-alpha** | 0 | 11.71 | 6.10 | 12.10 | 2.68 | 13.76 | 9.91 | 12.92 | 3.50 |
|  | 8 | 11.48 | 2.90 | 11.30 | 2.74 | 8.65 | 3.03 | 10.00 | 4.17 |
|  | 12 | 8.34 | N/A^a^ | –^b^ |  | 9.23 | 2.12 | –^b^ |  |
|  | 16 | 12.10 | 2.68 | 13.76 | 9.91 | 12.92 | 3.50 | 10.39 | 2.59 |

^a^One value.

^b^No value.

IFN, interferon; IL, interleukin; N/A, not applicable; SD, standard deviation; TNF, tumor necrosis factor.

**Supplementary Table S3.** Plasma fatty acid content in mice fed with mineral oil 30 µg/mouse/day or corn oil 30 µg/mouse/day for 16 weeks.

|  | Plasma fatty acid content, µmol/L | | | | | | | | | | | | | | | | |
| --- | --- | --- | --- | --- | --- | --- | --- | --- | --- | --- | --- | --- | --- | --- | --- | --- | --- |
|  | C14:0 | C16:0 | C16:1 | C18:0 | C18:1  cis | C18:2  cis | C18:3 n6 | C18:3 n3 | C20:0 | C20:1 n9 | C20:2 | C20:3 n6 | C20:4 n6 | C20:5 | C22:0 | C24:0 | C22:6 |
| **Control (day zero)** | | | | | | |  |  |  |  |  |  |  |  |  |  |  |
| Mean | 5.9 | 571.5 | 81.8 | 328.0 | 1364.0 | 394.6 | 7.0 | 2.6 | 2.6 | 18.2 | 27.1 | 27.0 | 275.3 | 2.3 | 0.9 | 0.9 | 62.9 |
| SD | 2.0 | 144.2 | 22.8 | 79.8 | 378.3 | 108.2 | 3.0 | 0.7 | 0.9 | 5.8 | 9.6 | 6.4 | 86.1 | 0.5 | 0.2 | 0.3 | 18.3 |
| **Corn oil 30 µL/mouse/day group** | | | | | | |  |  |  |  |  |  |  |  |  |  |  |
| Mean | 6.0 | 535.3 | 88.2 | 327.2 | 1383.3 | 389.2 | 6.1 | 2.1 | 2.8 | 16.7 | 26.1 | 25.7 | 275.2 | 1.7 | 1.1 | 1.2 | 43.6 |
| SD | 2.6 | 159.6 | 26.6 | 85.5 | 397.5 | 107.5 | 1.8 | 0.8 | 1.1 | 5.7 | 8.0 | 6.2 | 75.3 | 0.6 | 0.4 | 0.3 | 11.1 |
| **Mineral oil 30 µL/mouse/day group** | | | | | | |  |  |  |  |  |  |  |  |  |  |  |
| Mean | 5.2 | 494.6 | 88.6 | 286.3 | 1259.2 | 327.4 | 5.1 | 1.9 | 2.5 | 14.9 | 27.4 | 26.5 | 212.9 | 1.8 | 1.0 | 1.0 | 40.4 |
| SD | 3.1 | 201.1 | 31.7 | 107.5 | 464.2 | 135.9 | 2.3 | 1.2 | 1.2 | 7.1 | 9.3 | 7.1 | 89.4 | 0.9 | 0.3 | 0.3 | 21.1 |
| **Independent samples *t*-test** | | | | | | |  |  |  |  |  |  |  |  |  |  |  |
| Corn oil *vs* mineral oil, *p*-value | 0.38 | 0.44 | 0.96 | 0.15 | 0.32 | 0.09 | 0.10 | 0.49 | 0.41 | 0.35 | 0.58 | 0.65 | 0.01 | 0.58 | 0.36 | 0.18 | 0.50 |
| Corn oil *vs* control, *p-*value | 0.94 | 0.53 | 0.50 | 0.98 | 0.90 | 0.90 | 0.41 | 0.14 | 0.62 | 0.49 | 0.78 | 0.58 | 1.00 | 0.01 | 0.02 | 0.07 | 0.01 |
| Mineral oil *vs* control, *p-*value | 0.46 | 0.21 | 0.48 | 0.22 | 0.49 | 0.14 | 0.09 | 0.06 | 0.81 | 0.17 | 0.91 | 0.84 | 0.07 | 0.06 | 0.11 | 0.44 | 0.01 |

SD, standard deviation.

**Supplementary Table S4.** Hepatic fatty acid content in mice fed with mineral oil 30 µg/mouse/day or corn oil 30 µg/mouse/day for 16 weeks.

|  | Hepatic fatty acid content, µg/mg liver protein | | | | | | | | | | | | | | | | |
| --- | --- | --- | --- | --- | --- | --- | --- | --- | --- | --- | --- | --- | --- | --- | --- | --- | --- |
|  | C12:0 | C14:0 | C14:1 | C16:0 | C16:1 | C18:0 | C18:1 trans | C18:1 cis | C18:2 trans | C18:2 cis | C20:0 | C20:1 | C20:4n6 (ARA) | C20:5n3 (EPA) | C24:0 | C24:1 | C22:6n3 (DHA) |
| **Corn oil 30 µL/mouse/day group** | | | | | | |  |  |  |  |  |  |  |  |  |  |  |
| Mean | 0.36 | 2.9 | 0.11 | 95.8 | 12.9 | 31.6 | 0.12 | 326 | 0.04 | 35.5 | 0.86 | 6.4 | 17.9 | 0.05 | 0.33 | 0.82 | 1.7 |
| SD | 0.09 | 0.5 | 0.03 | 16.0 | 2.5 | 2.8 | 0.08 | 56 | 0.03 | 4.7 | 0.25 | 0.7 | 1.2 | 0.05 | 0.07 | 0.16 | 0.5 |
| **Mineral oil 30 µL/mouse/day group** | | | | | | |  |  |  |  |  |  |  |  |  |  |  |
| Mean | 0.32 | 3.0 | 0.05 | 101.0 | 18.3 | 28.5 | 0.08 | 345 | 0.04 | 22.4 | 0.41 | 5.5 | 13.8 | 0.06 | 0.25 | 0.46 | 1.7 |
| SD | 0.05 | 0.3 | 0.01 | 13.1 | 3.3 | 2.3 | 0.06 | 50 | 0.02 | 2.3 | 0.17 | 0.8 | 1.5 | 0.03 | 0.06 | 0.10 | 0.5 |
| **Depending on normality, independent samples *t*-test or Mann Whitney U test for independent samples** | | | | | | |  |  |  |  |  |  |  |  |  |  |  |
| *p* value | 0.38 | 0.73 | **< 0.01** | 0.51 | **< 0.01** | **0.03** | 0.33 | 0.50 | 0.90 | **< 0.01** | **< 0.01** | **0.04** | **< 0.01** | 0.82 | **0.04** | **< 0.01** | 0.98 |

ARA, arachidonic acid; DHA, docosahexaenoic acid; EPA, eicosapentaenoic acid; SD, standard deviation.

**Supplementary Table S5.** Mean ± SD dietary fatty acid content of mice fed with mineral oil 15 or 30 µg/mouse/day or fed with corn oil 15 or 30 µg/mouse/day for 16 weeks.

| Fatty acid | Dietary fatty acid content, mg/g diet | | | | | | | | | | | |
| --- | --- | --- | --- | --- | --- | --- | --- | --- | --- | --- | --- | --- |
|  | 15 µL/mouse/day corn oil | | | 30 µL/mouse/day corn oil | | | 15 µL/mouse/day mineral oil | | | 30 µL/mouse/day mineral oil | | |
| C12:0 | 0.037 | ± | 0.002 | 0.036 | ± | 0.003 | 0.038 | ± | 0.001 | 0.037 | ± | 0.007 |
| C14:0 | 0.25 | ± | 0.01 | 0.26 | ± | 0.01 | 0.25 | ± | 0.01 | 0.25 | ± | 0.02 |
| C14:1 | 0.009 | ± | 0.003 | 0.008 | ± | 0.001 | 0.010 | ± | 0.003 | 0.008 | ± | 0.003 |
| C16:0 | 37 | ± | 1 | 38 | ± | 1 | 37 | ± | 1 | 36 | ± | 2 |
| C16:1 | 0.33 | ± | 0.01 | 0.35 | ± | 0.01 | 0.33 | ± | 0.01 | 0.34 | ± | 0.03 |
| C18:0 | 51 | ± | 1 | 51 | ± | 1 | 51 | ± | 2 | 50 | ± | 3 |
| C18:1 trans | 0.005 | ± | 0.002 | 0.007 | ± | 0.002 | 0.005 | ± | 0.001 | 0.006 | ± | 0.002 |
| C18:1 cis | 49 | ± | 1 | 51 | ± | 1 | 48 | ± | 2 | 47 | ± | 2 |
| C18:2 trans | 0.007 | ± | 0.002 | 0.008 | ± | 0.003 | 0.007 | ± | 0.002 | 0.007 | ± | 0.002 |
| C18:2 cis | 10.9 | ± | 0.1 | 13.7 | ± | 0.4 | 9.0 | ± | 0.3 | 8.9 | ± | 0.5 |
| C18:3 | 0.34 | ± | 0.03 | 0.39 | ± | 0.02 | 0.32 | ± | 0.02 | 0.31 | ± | 0.03 |
| C20:0 | 1.6 | ± | 0.0 | 1.6 | ± | 0.0 | 1.6 | ± | 0.1 | 1.6 | ± | 0.1 |
| C20:1 | 0.10 | ± | 0.01 | 0.11 | ± | 0.01 | 0.10 | ± | 0.00 | 0.10 | ± | 0.01 |
| C20:5n3 (EPA) | 0.001 | ± | 0.000 | 0.001 | ± | 0.000 | 0.002 | ± | 0.001 | 0.002 | ± | 0.001 |
| C24:0 | 0.18 | ± | 0.01 | 0.19 | ± | 0.00 | 0.18 | ± | 0.01 | 0.17 | ± | 0.01 |
| C24:1 | 0.003 | ± | 0.000 | 0.003 | ± | 0.001 | 0.003 | ± | 0.000 | 0.003 | ± | 0.001 |
| C22:6n3 (DHA) | 0.027 | ± | 0.005 | 0.026 | ± | 0.006 | 0.026 | ± | 0.004 | 0.028 | ± | 0.007 |
| SAFA | 90 | ± | 2 | 91 | ± | 1 | 89 | ± | 3 | 88 | ± | 5 |
| MUFA | 50 | ± | 1 | 51 | ± | 1 | 48 | ± | 2 | 48 | ± | 3 |
| PUFA(n-3) | 0.36 | ± | 0.03 | 0.42 | ± | 0.02 | 0.34 | ± | 0.02 | 0.34 | ± | 0.04 |
| PUFA(n-6) | 11.0 | ± | 0.1 | 13.7 | ± | 0.4 | 9.0 | ± | 0.3 | 8.9 | ± | 0.5 |

DHA, docosahexaenoic acid; EPA, eicosapentaenoic acid; MUFA, monounsaturated fatty acid; PUFA, polyunsaturated fatty acid; SAFA, saturated fatty acid; SD, standard deviation.

**Figure legend**

**Supplementary Figure S1.** Mean (SD) plasma composition at 16 weeks of (**A**) PBMC, (**B**) T-cell subsets, (**C**) B-cell subsets, and (**D**) monocyte subsets. PBMC, peripheral blood mononuclear cell; SD, standard deviation.
